# Determinants of patient satisfaction and their willingness to return after primary total hip replacement: a cross-sectional study

**DOI:** 10.1186/s12891-016-1196-3

**Published:** 2016-08-08

**Authors:** Tom Schaal, Tonio Schoenfelder, Joerg Klewer, Joachim Kugler

**Affiliations:** 1 Department of Public Health, Dresden Medical School, University of Dresden, Loescherstrasse 18, 01309 Dresden, Germany; 2 Department of Public Health and Health Care Management, University of Applied Sciences Zwickau, Dr.-Friedrichs-Ring 2A, 08056 Zwickau, Germany

**Keywords:** Patient satisfaction, Willingness to return, Number of cases treated, Hospital, Doctor-patient relationship, Quality improvement, Joint replacement, Total hip replacement, Total hip arthroplasty, Expectations

## Abstract

**Background:**

Surveys of patient satisfaction and their willingness to return can be used for the optimization of processes, improving their quality, and increasing the satisfaction and loyalty in customers. This study looked at the factors significantly associated with patient satisfaction after primary total hip replacement (THR), and which affect the patients’ willingness to return to the same hospital for future treatment, even when unrelated to their THR.

**Methods:**

Data for the study was collected by written survey from 810 patients of 43 hospitals following their THR. Satisfaction and willingness to return were measured using a validated, multidimensional questionnaire, primarily based on six-point scales, which were then evaluated together with routine hospital data, according to bivariate and multivariate analyses.

**Results:**

The bivariate analysis showed a strong correlation between satisfaction or willingness to return and the health condition before hospitalization as well as the perceived length of stay. In contrast, the patient’s gender and the number of inpatient cases in a hospital with THR had no influence. The binary logistic regression analyses identified three predictors associated with overall satisfaction and seven predictors associated with willingness to return. The strongest factor for both dependent variables was the perceived length of stay, and the weakest factor for satisfaction was the treatment outcome.

**Conclusions:**

Overall, with all of the medical and service-related issues considered, high levels of satisfaction were reached. Despite the high satisfaction scores, probable causes for declining the willingness to return were identified. The results provide incentives for hospitals and medical professionals to attain a high satisfaction levels in their THR patients.

## Background

Patient expectations are becoming an increasingly important factor in current quality concepts, alongside the compliance with evidence-based guidelines [1]. Patient satisfaction represents an established indicator for measuring health care quality, and it is used by hospital management to monitor and improve service quality. From the patient’s perspective, overall satisfaction is also a good indicator of self-perceived health condition after surgery [2–4]. Patient satisfaction is multidimensional and provides the means to identify individual problem areas in the hospital and develop approaches for their solution [5]. Satisfaction can be understood as fulfillment of the needs and desires of the patients to a reasonable degree, and is rated differently by different individuals [6].

Total hip replacement (THR) is the treatment of choice for patients with severe, end-stage arthritis of the hip, and this will remain so in the near future [7]. THR represents a cost-effective method to alleviate pain in patients and to restore the function of the hip joint. More than one million hip replacements are performed each year around the world, and this number is expected to double during the next two decades [8]. Despite the predominantly high patient satisfaction after THR in recent years, between 3 and 16 % of patients are nevertheless dissatisfied [3, 9–11].

While pain, function, mental health, and sociodemographic information are frequently analyzed as indicators of satisfaction in this patient group, communicative aspects such as the effect of the patient information consultation have only been taken into account sporadically [4, 12–15]. Until now, the effects of interpersonal relationships in the medical arena and aspects of service quality on overall satisfaction have hardly been considered for hip replacement patients [16].

Chang et al. [16] examined service aspects as well as medical care in hospital-based joint replacement and came to the same conclusion as Ramaesh et al. [17] that hospitals, due to the heterogeneous patient structure, should identify the factors causing an increase in satisfaction of a plurality of patients.

In competitive health care systems, it is important for providers to have patients return for future treatments and not opt for other competitors [18]. Patient loyalty can be managed through the degree of satisfaction [19]. This involves identifying the factors that influence patients’ willingness to return to the same hospital, talk positively about it with others, and recommending it to friends [20]. Studies identifying the determinants of patients’ satisfaction and willingness to return to a hospital are relatively rare in the literature [21, 22].

In various studies, patient-reported treatment outcomes represented additional quality indicators in a number of inpatient cases in hospitals [23–25].

Therefore, the aim of this study was to identify the medical and service-related parameters and hospital characteristics significantly related to patient satisfaction and willingness to return after THR.

## Methods

### Patient data and patient recruitment

The study included randomly selected patients treated for THR in 47 hospitals of one federal state in Germany between 2010 and 2011. The population of this region was 3.06 million inhabitants (2011).

The survey was aimed at patients of five statutory health insurance providers with a market share of 78 % of the total population. Patient contact was secured by the health insurance provider and not by the hospitals, in order to ensure the uniformity of the questionnaire and exclude any directed patient selection. The questionnaire was based on a survey instrument developed by one of the health insurance providers and was drafted in German. This questionnaire was sent to the patients’ homes. Based on the performance data of the health insurance providers, patients were contacted that had been billed with a DRG (Diagnosis Related Group) for primary total hip replacement. In hospitals with more than 300 THR cases a year, 300 patients were chosen at random, whereas in hospitals with less then 300 cases, all patients were involved. Study participants were randomly selected on basis of age, sex, and the market share of their health insurance provider of the federal state where the study was conducted. In total, a maximum of 600 patients were contacted per hospital for the years 2010 and 2011. The survey was conducted between February and June 2012. At the time of the survey, the treatment of patients had been completed for 1–25 months. Participation was voluntary and anonymous.

Of 6,812 return postage-paid questionnaires mailed, 827 have been answered to and returned.

### Data collection

A validated questionnaire was used to determine patient satisfaction, sociodemographic information, and information about the hospital stay [26, 27]. Patient satisfaction was subdivided into one medical and one service-related battery of questions with 10 and 6 questions (Table 2), respectively, and was measured using a six-point scale (very good, good, satisfactory, adequate, inadequate, and unsatisfactory). The treatment outcome and the overall satisfaction in the hospital were also measured by using the same scale. For this purpose, patients were asked: If you were to evaluate the hospitalization received, how would you rate it? In addition, information was collected regarding the patients’ age (categorized at 10-year intervals, from 21 to >80), gender, and health condition prior to admission (excellent, good, fair, or poor). The questionnaire also ascertained the qualifications of the admitting physician, the perceived length of the hospital stay, and the occurrence of complications after discharge (Table 1). Finally, patients were asked whether they would choose the same hospital again (yes, no, or not sure). The willingness to return to the same hospital referred to treatment in the future of any kind, even not related to THR. Hospital characteristics regarding THR were obtained from the systematic hospital quality reports that each hospital is required to publish biannually. Items of particular interest in these reports were the number of THR inpatient cases, the indications for THR surgery, postoperative mobility (neutral-zero method), and whether there was any reoperative surgery during the period reported (yes/no). The neutral-zero method describes the range of motion of a joint in degrees of an angle around a certain axis.

**Table 1:** Effects of patient and hospital characteristics on overall satisfaction and willingness to return (*n* = 810)

	Variable	Value (n)	Overall satisfaction		Willingness to return		
			Satisfaction rate^d^	*p*-Value	Likely	Unlikely	*p*-Value
Data from the questionnaire	Sex	Male (314)	5.35	0.079^a^	281	29	0.237^c^
		Female (483)	5.28		427	54	
		No response	13		19		
	Age (years)	31–40 (1)	5.0	0.088^b^	1	0	0.294^c^
		41–50 (16)	5.36		11	4	
		51–60 (81)	5.32		74	7	
		61–70 (240)	5.36		214	25	
		71–80 (374)	5.31		335	35	
		>80 (95)	5.14		81	13	
		No response	3		10		
	Number of prior hospital stays^e^	1–2 (586)	5.35	0.002^b^	537	46	<0.001^c^
		3–5 (182)	5.2		150	29	
		>5 (24)	5.0		19	5	
		No response	18		24		
	State of health prior to hospitalization	Excellent (8)	5.5	0.002^b^	8	0	0.455^c^
		Good (136)	5.3		121	13	
		Fair (279)	5.21		243	34	
		Poor (371)	5.39		335	34	
		No response	16		22		
	Source of referral	General practitioner (90)	5.31	0.069^b^	79	10	0.058^c^
		Specialist (642)	5.33		580	59	
		Self-referral (12)	5.45		11	1	
		Emergency (39)	5.0		28	9	
		Transferred from other hospital (2)	5.5		2	0	
		No response	25		31		
	Length of stay	1–2 days (2)	5.0	0.478b	2	0	0.097c
		3–7 days (95)	5.29		84	11	
		1–2 weeks (571)	5.32		516	51	
		>2 weeks (131)	5.24		108	21	
		No response	11		17		
	Assessment of length of stay	Absolutely appropriate (614)	5.4	<0.001^b^	573	38	<0.001^c^
		Could have been longer (99)	4.92		74	23	
		Could have been shorter (12)	4.67		8	4	
		I cannot judge (77)	5.05		56	19	
		No response	8		15		
	Self-reported complications after discharge	Yes (99)	4.8	<0.001^c^	59	40	<0.001^c^
		No (693)	5.36		648	42	
		No response	18		21		
Data from the systematic hospital quality reports	Number of cases treated by hospital	High (9)^f^	5.35	0.021^c^	377	41	0.529^c^
		Low (34)^f^	5.26		342	43	
		Median (range)	299 (7–569)				
	Postoperative mobility (percentage of cases treated)	High (23)^f^	5.33	0.548^c^	354	32	0.053^c^
		Low (20)^f^	5.29		365	52	
		Median (range)	99.65 (84–100 %)				
	Indication for THR surgery (percentage of cases treated)	High (21)^f^	5.33	0.125^c^	359	43	0.827^c^
		Low (22)^f^	5.28		360	41	
		Median (range)	96.83 (65–100 %)				
	Reoperation during study period	Yes (35)^f^	5.3	0.161^c^	613	76	0.195^c^
		No (8)^f^	5.33		106	8	

^a^Mann-Whitney U test. ^b^Kruskal-Wallis test. ^c^Chi-squared test. ^d^Grouped median. ^e^Within the prior 5 years. ^f^Number of hospitals, not the sum of patients treated

### Statistical analysis

The descriptive statistics and frequencies were calculated. For data analysis, the six-point grading scales coded the best rating as six and the worst rating as one. The significance level for the entire study was *p* < .05. The data was analysed using SPSS software, version 20.0 (SPSS Inc., Chicago, IL, USA).

#### Bivariate analysis

Satisfaction scores showed a left-skewed distribution for the better rating, which is why non-parametric tests were used. Based on the overall satisfaction and willingness to return to the hospital, the analysis examined small cell scores for potential differences in patient-related and hospital-related variables by using the chi-square test or Fisher’s exact test, as well as multiple group comparisons by using the Kruskal-Wallis test. Hospital-related parameters regarding the number of inpatient cases of THR and indication for THR surgery were dichotomized (median split) for further study. The Man-Whitney U test was used for the medical and service-related batteries of questions and for the assessment of the treatment outcomes. Here, the ratings were divided into “satisfied” (very good, good) and “dissatisfied” (satisfactory-inadequate) and the willingness to return into “likely” (yes) and “unlikely” (no, not sure).

#### Multivariate analysis

Two separate binary logistic regressions with inclusion processes were chosen as a multivariate analysis method, for which the non-significant variables from the bivariate analysis were excluded [2]. This approach was chosen in order to obtain a simple model with some few degrees of freedom based on the sample size. The dependent variables were ‘overall satisfaction’ and ‘willingness to return’, which were graphed dichotomously as “satisfied” and “dissatisfied” and as “likely” and “unlikely”, respectively. The missing values of all independent variables were replaced by multiple imputation (iterative Markov chain Monte Carlo method, 10 iterations) for the logistic regression calculation [28–30]. The question regarding the doctor’s knowledge of the patient’s case history and course of disease had the highest missing rate, at 3.58 %.

## Results

Patients were excluded if the question of overall satisfaction remained unanswered or if no routine data were available for the respective hospital [31]. In all, 810 questionnaires from 43 hospitals were evaluated.

More than half of the study population was female (60.6 %), 71–80 years old, and had been to the hospital one to two times in the five years before the THR. Of all patients, 79.3 % were admitted to the hospital by a specialist, 11.1 % by a general practitioner, 1.5 % by self-admission, 4.8 % due to emergency, and 0.2 % by transfer from another hospital. Of the respondents, 45.8 % rated their health condition prior to hospitalization as poor, 34.4 % as fair, 16.8 % as good, and 1 % as excellent. A good two-thirds of patients had a hospital stay of 1 to 2 weeks. The majority of participants (75.8 %) classified the duration of stay in the hospital as reasonable, while 12.2 % found that it could have been longer and 1.5 % indicated that it could have been shorter; 9.5 % were unsure. Of the patients, 88.7 % were satisfied with the treatment outcome (very good – good), and 11.3 % were dissatisfied (satisfactory-inadequate). Postoperative complications reported on the patients’ own accounts (self-reported) in the questionnaire were found in 12.2 % of the study participants. The number of of THRs performed in 2010 in each hospital was between 7 and 569. The existence of an indication to perform this surgery, based on clinical, laboratory, or radiological findings, varied according to hospital to be between 64.7–100 %. In this context, reimplantations were recorded in 33 of 43 hospitals, and death occurred in 11 institutions (Table 1).

### Satisfaction scores

Of all patients, 88.8 % would choose to be treated again in the same hospital, 2.1 % ruled this out, and 8.3 % were not sure. Of all study participants, 737 (91 %) rated their overall hospital stay as very good or good (grouped median: 5.31). Patients were most satisfied with the friendliness of the nursing staff (5.5) and the clear information provided about the anesthesia (5.5), followed by the friendliness of the doctors (5.49). The worst ratings were given as regards to the room equipment (5.11) and regarding the clear information provided about the medicines to take (5.07).

### Bivariate analysis

In the bivariate analysis, the 16 criteria of the medical and service-related batteries of questions showed a statistically significant (*P* < .001) influence on the patients’ overall satisfaction and willingness to return (Table 2). Whereas, the number of previous hospitalizations in the last 5 years (*P* < .05), the perceived length of hospital stay (*P* < .001), self-reported complications after discharge from the hospital (*P* < .001), as well as the treatment outcome (*P* < .001), were all associated with both dependent variables. A difference in responses given between the health condition prior to hospitalization (*P* < .05) and the number of inpatient cases with THR (*P* < .05) could only be established with the overall satisfaction (Table 1). Satisfaction and willingness to return decreased as previous hospitalizations increased, from 1–2 visits (5.35/92.1 %), to 3–5 visits (5.2/83.8 %), to more than five visits (5.0/79.2 %). Patients who considered their stay to be reasonable were more satisfied (5.4) and had a higher willingness to return (93.8 %) than patients who considered their stay too short (4.92/76.3 %) or too long (4.67/66.7 %), or were not sure (5.05/74.7 %). Respondents with self-reported complications after discharge were less satisfied (4.8) and less willing to return (59.6 %) than participants without these complications (5.36/93.9 %). While patients who considered themselves in excellent health prior to hospitalization were the most satisfied (5.5), the ratings of participants with poor health (5.39) were better than those of participants with fair (5.21) and good health (5.3). Patients were more satisfied (5.35) in hospitals with large sample sizes than in hospitals with smaller sample sizes (5.26). Indication for THR surgery, postoperative mobility, and reoperation during the treatment period had no influence on the overall satisfaction or willingness to return. The same was true for the patient’s gender, age, type of hospital admission, and length of stay (Table 1).

**Table 2:** Individual assessment of satisfaction and willingness to return on items related to medical care and service received (grouped median)

Satisfaction criterion^a^	Overall satisfaction		Willingness to return	
	Satisfied patients^b^	Dissatisfied patients^c^	Likely	Unlikely^d^
Organization of hospital admission	5.51	4.88	5.51	5.03
Doctor’s knowledge of medical history and course of the disease	5.41	4.81	5.41	4.90
Clear physician answers to patient questions	5.44	4.66	5.44	4.80
Assessment of medical care received	5.40	4.24	5.39	4.57
Clear explanation of surgery	5.55	4.55	5.54	4.84
Clear explanation of anesthesia	5.56	4.81	5.54	5.06
Clear explanation of medications to be taken	5.15	4.0	5.14	4.33
Organization and conduct of tests	5.40	4.58	5.39	4.77
Privacy during testing	5.39	4.73	5.38	4.93
After-discharge preparations	5.27	3.86	5.27	4.18
Friendliness of the nursing staff	5.57	4.63	5.56	4.82
Friendliness of the doctors	5.55	4.69	5.55	4.86
Friendliness of other hospital staff	5.39	4.66	5.39	4.77
Room amenities	5.17	4.48	5.17	4.58
Cleanliness	5.43	4.69	5.44	4.78
Quality of food	5.27	4.58	5.27	4.70
Treatment outcome	5.47	4.25	5.48	4.32

^a^Difference between satisfied/dissatisfied patients and likely/unlikely was significant. *P* < .001 Mann-Whitney U test

^b^Overall satisfaction ranked very good or good

^c^Overall satisfaction ranked satisfactory, adequate, inadequate, dissatisfactory

^d^Willingness to return ranked no or do not know

### Multivariate analysis

**Fig. 1:**
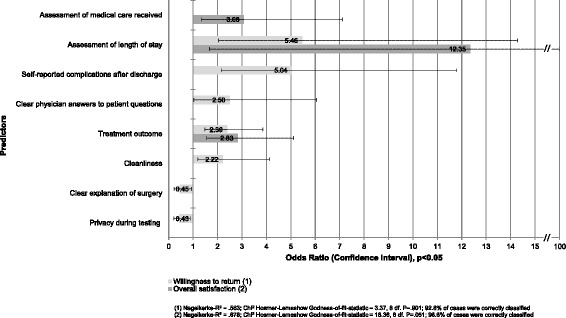
Predictors asociates with overall satisfaction and willingness to return (logistic regression)

The multivariate analysis revealed three predictors associated with overall satisfaction and seven variables associated with willingness to return (Fig. 1). While medical variables and postoperative parameters affected both dependent variables, service aspects were only associated with the patient’s willingness to visit the hospital again. Patients who rated their perceived length of stay as reasonable were the strongest factor influencing overall satisfaction in relation to a stay that was too long (OR [Odds Ratio]: 12.35), followed by the rating of the medical care (OR: 3.08). Willingness to return was most strongly influenced by the reasonableness of the length of stay (OR: 5.64), compared to patients who could not judge the length of stay or who had self-reported complications (OR: 5.04) [32]. Clear information provided about the surgery (OR: 0.45) and the protection of privacy (OR: 0.43) had a negative effect on patients’ willingness to return to the same hospital. Routine hospital data concerning the number of inpatient cases of a hospital with THR, indication for THR surgery, postoperative mobility, and reoperation during the treatment period could not be sufficiently protected against randomness and had no significant influence on the dependent variables.

## Discussion

This study examined a sample of 810 patients after undergoing THR in 43 German hospitals. The main objective was to identify differences between the variables predicting overall patient satisfaction and those predicting willingness to return to the hospital. Consistent with previous study results, overall satisfaction and willingness to return were found to represent different constructs that do not measure the same thing, and that the majority of patients are satisfied [3, 9–11, 22, 33, 34]. Previous research investigating overall satisfaction and willingness to return had not considered hip replacement patients. Because of the limited research on this topic, the seven predictors of willingness to return produced the initial findings for this population studied [21, 22]. The number of inpatient cases with THR, as well as all included routine hospital data, had no significant effect on the overall satisfaction or willingness to return to the same hospital in the multivariate analysis, and were therefore not suitable as quality indicators in this regard. The preclinical patient characteristics were also not associated with the two dependent variables and were found to conform to earlier studies [35].

### Key findings

At 91 %, patient satisfaction was comparable to previous study results [3, 9–11]. At 88.8 %, willingness to return to the hospital for further treatment slightly exceeded the values seen in previous studies, which were between 78.9 and 86.7 % [21, 23, 24].

While all items in the medical and service-related battery of questions, as well as some patient-related variables, were associated with overall satisfaction and willingness to return in the bivariate analysis, this relationship could not be maintained in the multivariate analysis. The multivariate results of this study show that some aspects of hospitalization influence the patients’ overall satisfaction, as well as the likelihood that they will return to the hospital. These were the length of stay, which was also the strongest factor in both logistic regressions, and the treatment outcome, which can be understood as an expression of the physical activity recovered and the reduction of functional limitations and pain [36, 37]. Patients were questioned about the actual length of stay, as well as the perceived length of stay, because the medical records were not available from the respective hospitals. Finkelstein et al. [38] and Husted et al. [39] likewise came to the conclusion that a hospital stay perceived as too long leads to lower satisfaction, whereby Husted et al. also found that this factor had the largest effect on satisfaction. A probable cause may be rising patient expectations for a shorter length of stay as a consequence of an accelerated rehabilitation after hip replacement [12, 40]. In addition, the patients’ lack of knowledge of individual factors, which may influence the length of stay, leads to false expectations. An appropriate length of stay can be derived by considering comorbidities, or a complicated diagnosis based on the average length of hospital stay of the DRG for THR, and can serve the patient as an orientation in the context of patient education. These results are not transferable to the willingness to return, because no conclusions are possible about the patient group that answered the length of stay question with ‘I cannot judge’.

This is offset by parameters that cause an increase in satisfaction but no willingness to return, and vice versa. The assessment of medical care was associated with overall satisfaction, and this supports the conclusion that the perception of the interpersonal relationship in the medical arena has a positive influence on patient satisfaction [16, 18]. Service-related issues did not affect satisfaction. Apart from various factors that affected both dependent variables, the study showed that high ratings for clear information provided about the surgery and the protection of privacy were associated with a decreased likelihood of returning to the hospital, contrary to the assumption that satisfied patients are automatically loyal customers. Evidence-based patient decisions, as part of participatory decision-making between doctors and patients, are characterized by a high level of satisfying information. This may lead to a conflict of interest; for example, in the choice of a fixation option for the hip replacement (cementless, cemented, hybrid, or reverse hybrid), which decreases the willingness to return to the same hospital for treatment [8, 41–43]. While high satisfaction levels with regard to privacy indicate that it is being taken into account, this seems to be paradoxical in view of the use of shared patient rooms [44]. What is meant is that patients, despite the lack of retreat possibilities in shared patient rooms, e.g., during doctor visits or visits by relatives, assess the privacy as positive. Furthermore, privacy is perceptibly disturbed prior to surgery, due to intense feeling of shame resulting from the uncovered genital and gluteal areas, and can be a cause of decreasing willingness to return, in spite of otherwise positively perceived privacy issues [45]. In contrast to the overall satisfaction, there was an influence of room cleanliness on the willingness to return. Patients can qualitatively assess this service aspect, in contrast to medical knowledge, and they use it as a surrogate indicator to establish a connection with the perceived medical treatment and correct diagnosis, which may have an effect on whether or not the patient returns to the same hospital for treatment [18, 46]. The present study supports the findings of Anakwe et al. [10], which demonstrate that postoperative complications cannot predict dissatisfaction but are associated with the willingness to return. Patients suffering no complications after being discharged from the hospital are more likely to return to this hospital if further treatment is required.

Despite the age of the data of 5 years at the time of evaluation, the results are considered relevant. Currently, ceramic implants and new plastic compounds are more frequently used, instead of the previous metal implants used for treatment in the period of 2010–2011. Despite all the advantages of these new implant methods, infections, fracture around the implant, difference in leg length, or dislocation, can still lead to complications, which may also influence the willingness to return [47, 48]. The age distribution of patients was solid in the age groups from 2010 to 2014, with minimal variation. Therefore, it is not expected that the determining factor ‘age’ has an influence on the results [49, 50].

The predictors identified represent relevant steps throughout the care process from the patient’s perspective and provide incentives for effective measures [5] as to how hospital management and health professionals can influence patient satisfaction and customer loyalty. The multicenter study approach of 43 hospitals produced hospital-independent findings and added to the previous state of research [9, 17].

### Limitations

Several limitations should be considered while interpreting the results of this cross-sectional study. First, the information on non-participants is not known. Emberton & Black noted that the non-respondents in satisfaction surveys dealing with surgery are often older and in a worse health condition than the respondents, whereby the error of these people overestimating positive results and underestimating negative effects is considered low [51]. According to the findings of Polk et al., overall satisfaction is not influenced by the tendency toward worse satisfaction results among non-respondents [52]. Potential non-response bias and their resulting limitations on the study results cannot be excluded, because the influence of non-participants could not be controlled, and the net rate of return was very low at 11.9 %. Perhaps patients would have been more likely to participate in the survey if it were close to the treatment. The majority of participants were older people, for whom functional limitations may exist and make it difficult to understand or fully complete the questionnaire. A follow-up action by reminders or telephone queries could lead to a higher response rate, although it may be suspected that this will not comply with the anonymity assured. Second, while the sample of patients from 43 hospitals does approximate the hospital structure of a region of Germany, the capability to generalize these results to fit other regions and countries still needs to be clarified. Third, the recall bias because of the time interval between treatment in 2010 and the questionnaire survey in 2012 cannot be excluded, e.g., with regard to self-reported complications.

Nevertheless, with an explained variance of 67.8 or 56.3 % using Nagelkerke R^2^, overall satisfaction is well predicted by the independent variables, and Hosmer-Lemeshow tests showed an adequate goodness of fit [53]. In contrast to conventional methods, the multiple imputation enabled asymptotically unbiased estimates of the missing values [54].

## Conclusions

The results showed that variables influenced by medical professionals, as opposed to service components, had the greatest effect on satisfaction and willingness to return to the hospital. The length of stay was the strongest factor on these independent variables, and this illustrates their importance in view of the projected doubling of THR cases in the years to come, with simultaneously decreasing bed counts. Improving patient education by medical professionals about individual factors affecting the length of stay can help ensure that it is no longer perceived as too long or unrateable. Since this surgery often involves re-treatment of the second joint, high scores in satisfaction and willingness to return are especially important for retaining the same patients. Further studies are needed to take account of the long-term results of the patient population studied here.

## Abbreviations

CI, confidence interval; DRG, Diagnosis Related Groups; OR, odds ratio; SPSS, statistical package for the social sciences; THR, total hip replacement
